# Sex- and age-dependent outcomes of 9-hour time-restricted feeding of a Western high-fat high-sucrose diet in C57BL/6J mice

**DOI:** 10.1016/j.celrep.2021.109543

**Published:** 2021-08-17

**Authors:** Amandine Chaix, Shaunak Deota, Raghav Bhardwaj, Terry Lin, Satchidananda Panda

**Affiliations:** 1 Salk Institute for Biological Studies, La Jolla, CA 92037, USA; 2 Present address: Department of Nutrition and Integrative Physiology, University of Utah, Salt Lake City, UT, USA; 3 Lead contact

## Abstract

Time-restricted feeding (TRF) is a nutritional intervention wherein food intake is limited to a consistent 8- to 10-h daily window without changes in nutritional quality or quantity. TRF can prevent and treat diet-induced obesity (DIO) and associated metabolic disease in young male mice fed an obesogenic diet, the gold standard preclinical model for metabolic disease research. Because age and sex are key biological variables affecting metabolic disease pathophysiology and response to therapies, we assessed their impact on TRF benefits by subjecting young 3-month-old or middle-aged 12-month-old male and female mice to *ad libitum* or TRF of a Western diet. We show that most of the benefits of TRF are age-independent but are sex-dependent. TRF protects both sexes against fatty liver and glucose intolerance while body weight benefits are observed only in males. We also find that TRF imparts performance benefits and increases survival to sepsis in both sexes.

## In brief

Chaix et al. show that time-restricted feeding (TRF) can improve metabolic functions in an age-independent but sex-dependent manner. While TRF of a Western diet blunted weight gain and adipose tissue inflammation in males only, it protected against fatty liver disease and glucose intolerance in both sexes.

## Graphical Abstract

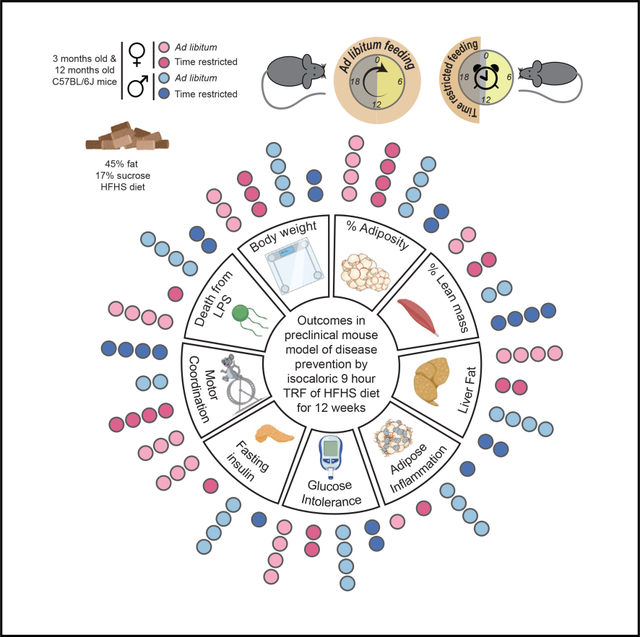

## INTRODUCTION

Sex and age modulate both the impact of an obesogenic diet on metabolic health and the outcomes of interventions for metabolic diseases. Females are more likely to gain fat mass and resist net fat loss. In fact, the global prevalence of obesity is higher in women than in men (Kelly et al., 2008). Females have more fat and less lean mass relative to males, yet for equivalent adiposity, females are more insulin sensitive than males. Epidemiological and clinical studies have demonstrated major sex differences in the prevalence of metabolic disorders, with women being protected from cardiovascular disease (CVD) relative to men before menopause. Despite this lower risk, in the last 20 years, CVD risk has increased in pre-menopausal women, which has been attributed to an increase in the prevalence of diabetes that can offset this sexual dimorphism (Regensteiner et al., 2015). Once females develop metabolic diseases, such as fatty liver disease, they are prone to progress faster to more severe forms. Age is also a major biological variable in metabolic health. There is an increased tendency to accumulate adiposity in middle age, which alone or in combination with older age elevates the risk for metabolic diseases.

Although age and sex significantly modulate metabolic health, most preclinical metabolism research is done on young male mice. This is partly due to earlier observations that young female mice of the widely used C57BL/6 strain fed a high-fat diet (HFD) are less prone to diet-induced obesity (DIO) and associated metabolic diseases than are male mice. The female mice are leaner, do not develop hyperinsulinemia, and display less adipose tissue inflammation (Grove et al., 2010; Pettersson et al., 2012) than do the male mice. Estrogen depletion experiments (ovariectomized females or post-menopausal models) (Hong et al., 2009; Riant et al., 2009; Zhu et al., 2013) and estrogen replacement (E2 treatment) (Riant et al., 2009) strongly support the idea that estrogen signaling underlies sexual dimorphism in metabolic and inflammatory responses between males and females. Recent studies have shown that C57BL/6J (B6) female mice fed a HFD become glucose intolerant, and long-term feeding of a HFD leads to similar cardiometabolic dysfunction in both male and female C57BL/6J mice (Bruder-Nascimento et al., 2017).

Time-restricted feeding (TRF) is a nutritional intervention strategy in which mice are fed within a consistent 8- to 12-h interval daily. A TRF cohort typically consumes isocaloric food as their *ad libitum*-fed (ALF) counterparts and yet exhibit significant reduction in weight gain and metabolic diseases that are found in the ALF cohort (Chaix et al., 2014; Delahaye et al., 2018; Duncan et al., 2016; Hatori et al., 2012; Sherman et al., 2012; Sundaram and Yan, 2016; Woodie et al., 2018). TRF impacts multiple metabolic organs including but not limited to liver, muscle, and adipose tissues. In addition, lifelong TRF on a normal chow diet can increase longevity in male mice (Mitchell et al., 2019). This raises the possibility that TRF can be beneficial in females as well. Accordingly, recent studies showed that TRF can (1) delay mammary tumor progression in HFD-fed female mice (Sundaram and Yan, 2018) and (2) provide protection from DIO, fatty liver, and insulin resistance in postmenopausal or ovariectomized C57BL/6J mice (Chung et al., 2016; Omotola et al., 2019). An experimental paradigm in which TRF and ALF cohorts consume isocaloric diet offers a clinically relevant model to assess both the impact of an obesogenic diet on disease and an isocaloric intervention on both sexes and at different ages.

Recently, a series of pilot human studies have shown that time-restricted eating (TRE) can be an effective behavioral intervention to reduce the burden of metabolic diseases, yet these smaller studies are not powered enough to identify the effects of age or sex on clinical outcomes (Gabel et al., 2018; Gill and Panda, 2015; Sutton et al., 2018; Wilkinson et al., 2019).

In this study, we tested the effects of 3 months of ALF or TRF of a Western diet (WD) on body composition, metabolic health, and overall fitness in young and middle-aged male and female mice. Extensive metabolic phenotyping as well as performance and cognitive assays were conducted in parallel in young (3 months old) and middle-aged (12 months old) male and female C57BL/6J mice. We found in male mice that TRF prevented weight gain and improved health relative to ALF at both ages. Although female mice under TRF were not protected from weight gain, they showed several health benefits relative to their ALF counterparts and were even protected from lipopolysaccharide (LPS)-induced lethality. Thus, TRF can delay metabolic dysfunction and promote healthy aging in middle-aged pre-clinical male and female mouse models of metabolic disease.

## RESULTS AND DISCUSSION

### TRF prevents body weight gain only in male mice

**Figure 1.**
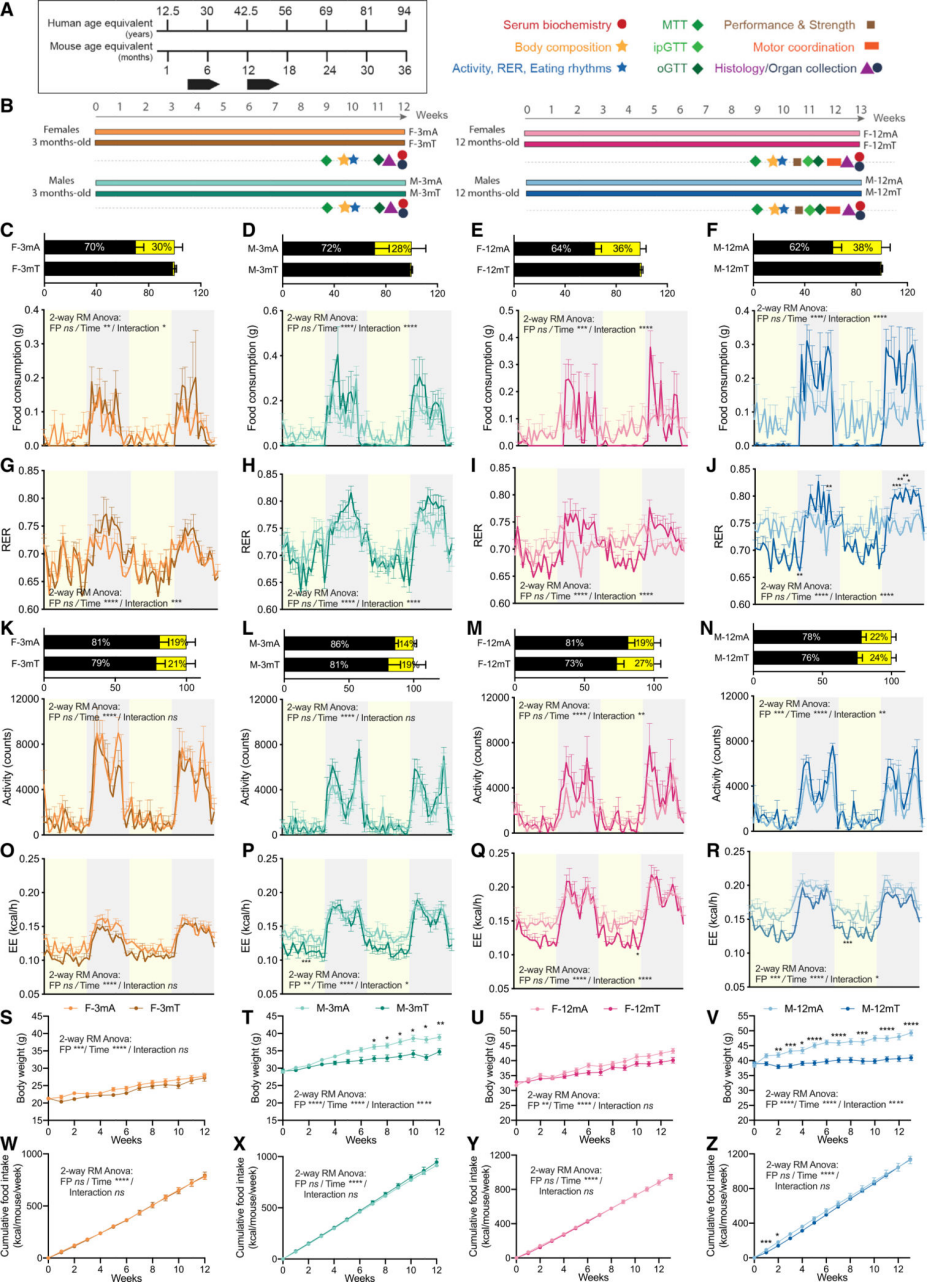
Isocaloric time-restricted feeding prevents significant weight gain in male mice (A) Schematic of human/mouse age equivalent. (B) Schematic of the experimental design. (C–F) Daily percentage of food consumption during the dark and light phase and food consumption during 48 h of recording in metabolic chambers in 3-month-old females (C) and males (D), and 12-month-old females (E) and males (F) after 10 weeks of intervention (n = 5–6/group). (G–J) RER during 48 h of recording in metabolic chambers in 3-month-old females (G) and males (H), and 12-month-old females (I) and males (J) after 10 weeks of intervention (n = 5–6/group). (K–N) Daily percentage of activity during the dark and light phase and activity during 48 h of recording in metabolic chambers in 3-month-old females (K) and males (L), and 12-month-old females (M) and males (N) after 10 weeks of intervention (n = 5–6/group). (O–R) Energy expenditure during 48 h of recording in metabolic chambers in 3-month-old females (O) and males (P), and 12-month-old females (Q) and males (R) after 10 weeks of intervention (n = 5–6/group). (S–V) Evolution of body weight in 3-month-old females (S) and males (T) (n = 20/group), and 12-month-old females (U) and males (V) during the 12/13 weeks of intervention (n = 50/group). (W–Z) Evolution of cumulative food consumption in 3-month-old females (W) and males (X) (n = 20/group), and 12-month-old females (Y) and males (Z) during the 12/13 weeks of intervention (n = 50/group). Statistics: (C–Z) Two-way repeated-measures ANOVA (factors: feeding paradigm [FP] and time; inset) and Sidak’s multiple comparisons tests (on graph). Data are presented as mean ± SEM with result of statistical test, with *p < 0.05, **p < 0.01, ***p < 0.001.

To test the impact of sex and age on energy metabolism and body weight (BW) regulation, we used four experimental cohorts: male and female C57BL/6J mice 3 months old (equivalent to ~20-year-old humans) and 12 months old (equivalent to ~42 year-old-humans) at the start of the feeding protocol (Figure 1A). Mice were fed a Western diet rich in both fat and sucrose (Research Diet D12451, 45% fat, 17% sucrose). ALF cohorts had unrestricted access to food throughout the 12-h light/12-hdark cycle, while the TRF cohorts had access to food only for 9 h daily during the dark phase (zeitgeber time [ZT]13–ZT22, where ZT0 denotes light ON) 7 days/week for 12–13 weeks. Metabolic and fitness assays were performed after at least 10 weeks into the feeding protocols (Figure 1B).

Monitoring of daily eating patterns in a subset of mice in indirect calorimetry chambers (Figures 1D–1F) revealed that male mice displayed the characteristic disrupted eating pattern of mice fed a HFD. As previously shown (Chaix et al., 2014, 2019; Hatori et al., 2012; Kohsaka et al., 2007), the young male mice increased their food intake during the light/inactive phase and consumed nearly 28% of food during the light phase, which further increased to 38% in older males (Figures 1D and 1F). Similar results were obtained in females with 30% of the daily intake in the light phase in 3-month-old females on ALF (F-3mA) and 36% in 12-month-old females on ALF (F-12mA) (Figures 1C and 1E). Two-way repeated-measured ANOVA of food consumption data revealed that the temporal feeding pattern was significantly different between mice on ALF and TRF in all four groups (interaction term significant in all groups; Figures 1C–1F; Table S1). Two-way ANOVA revealed that age significantly affects the daily percent of food consumption during the light phase (Figure S1J; Table S1). In all, these results suggest that a Western diet disrupts eating rhythms in both males and females, and that the proportion of food consumed during the day increases as mice age. Regardless of those differences in daily partitioning of food intake, total daily food consumption was equivalent between the ALF and TRF arms of each cohort, and was lower in females than in males (Figures 1C–1F and 1W–1Z; Figures S1F–S1I).

The daily rhythm in substrate usage was measured by indirect calorimetry. Two-way repeated-measures ANOVA of the respiratory exchange ratio (RER) showed a significant difference between mice on ALF and TRF in all four groups (interaction term significant in all groups; Figures 1G–1J; Table S1). TRF was associated with better partitioning of substrate usage as indicated by higher RER values during the dark phase and lower values during the light phase in TRF compared to ALF (Figures 1G–1J). This difference was even more pronounced in 1-year-old mice, as the RER flattens with aging under ALF but not under TRF. The average amplitude differences between the dark and light phases values of the RER were 0.024 in F-3mA compared to 0.046 in 3-month-old females on TRF (F-3mT), 0.037 in 3-month-old males on ALF (M-3mA) compared to 0.068 in 3-month-old males on TRF (M-3mT), 0.004 in F-12mA compared to 0.048 in 12-month-old females on TRF (F-12mT), and 0.007 in 12-month-old males on ALF (M-12mA) compared to 0.07 in 12-month-old males on TRP (M-12mT) (Figures 1G–1J). Activity (Figures 1K–1N) and energy expenditure (EE) (Figures 1O–1R) recordings in metabolic cages for 48 h showed the expected daily rhythms. Two-way repeated-measures ANOVA of activity data revealed that the temporal profile of activity was significantly different in old but not in young mice on ALF and TRF (interaction term in Figures 1K–1N; Table S1). Within each group, there was no difference in activity in young males and females and 1-year-old females between ALF and TRF, but a significant increase of activity in 1-year-old males on TRF was observed (Figures 1K–1N; Figures S1A–S1D). Finally, we observed a significant general decrease in activity between young and old females but not in males (Figure S1E). Two-way repeated-measures ANOVA revealed that EE was significantly affected by the feeding paradigm (FP) term in males but not in females irrespective of age (Figures 1O–1R; Table S1).

At the start of the feeding experiment, 3-month-old animals weighed less than 12-month-old ones, with females being lighter than males irrespective of age (average weights: 21.3 g, F-3m; 29.1 g, M-3m; 32.3 g, F-12m; and 38.8 g, M-12m) (Figures 1S–1V). TRF significantly prevented BW gain in both 3-month-old and 1-year-old males (Figures 1T and 1V). After 12 weeks on TRF, M-3mA gained 9.9 g (34% BW gain) whereas M-3mT gained only 5.6 g (19% BW gain). TRF protection against BW gain was even greater in 1-year-old males, with M-12mA gaining 10.7 g (28% BW gain) and M-12mT gaining only 2 g (5% BW gain) after 12 weeks on the feeding regimen. In contrast, TRF had no significant effect on weight gain in female mice (Figures 1S and 1U). F-3mA gained 6.6 g (31% BW gain), and F-3mT gained 5.9 g (28% BW gain). F-12mA gained 11.6 g (36% BW gain) and F-12mT gained 7.3 g (22% BW gain). According to JAX BW information for aged C57BL/6J mice, on a normal chow diet, the % BW gain from 52 to 64 weeks (12 weeks) is approximately 8% for males and 8.6% for females. In our study, despite the initial difference in body weight, we observed a similar % BW gain on a Western diet *ad libitum* between middle-aged males and females (28% for males, 34% for females). Thus, *ad libitum* consumption of this Western diet leads to increased weight gain in both males and females.

Weekly monitoring of food intake showed no difference between food consumption in ALF and TRF males irrespective of age (Figures 1X and 1Z). Thus, the protective effect of TRF against BW gain in males was independent from changes in energy intake. These BW benefits are not seen in females, revealing a sex-dependent response to TRF. Importantly, the lack of protection in females could not be attributed to lack of BW gain, as females on ALF gained as much % BW as males.

### TRF protects from fatty liver in both sexes while reduction in adipose inflammation and serum cholesterol levels are only seen in males

**Figure 2.**
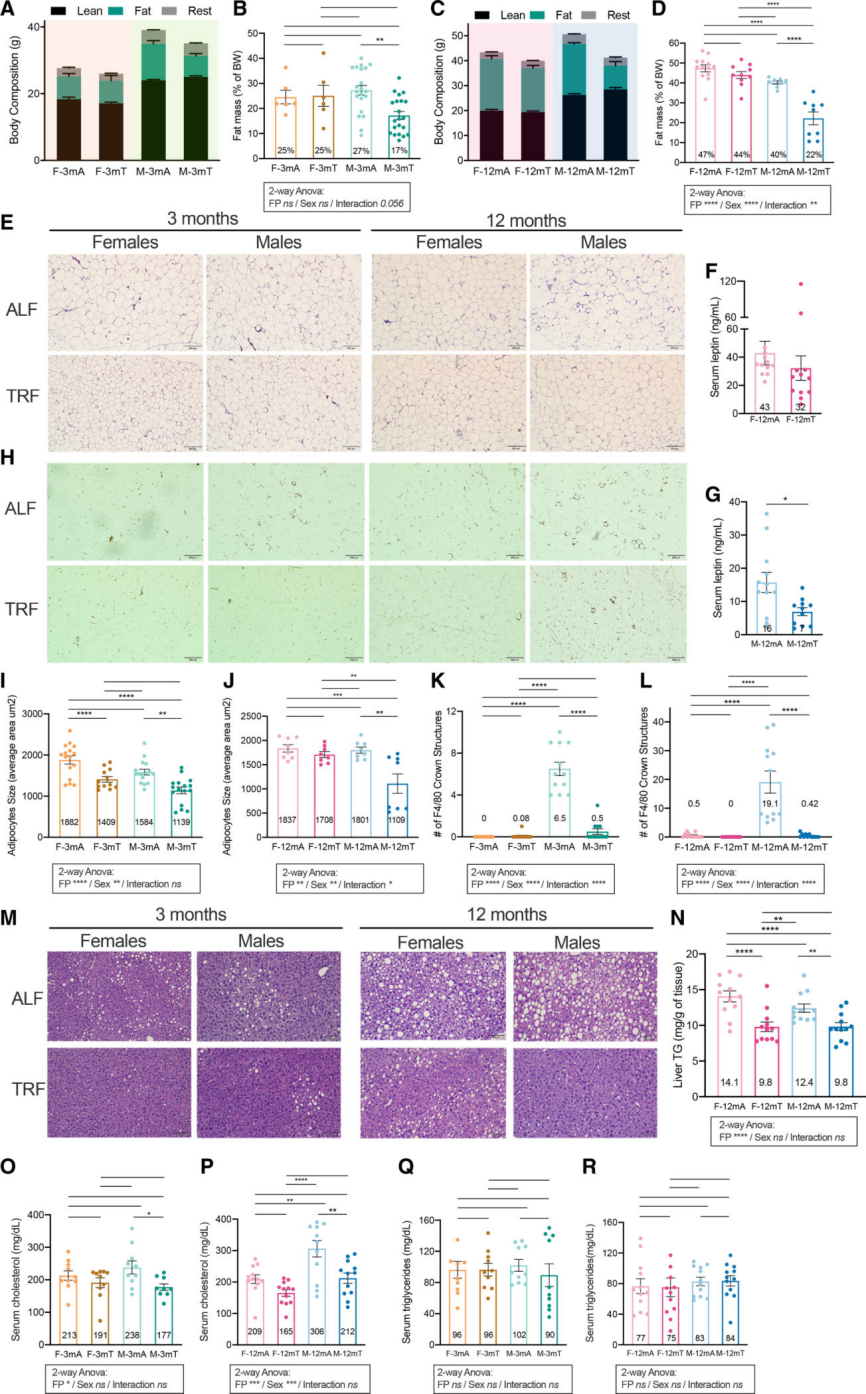
Shared and sex-specific effects of TRF on adiposity, adipose tissue inflammation, and liver fat (A–D) Body composition after 10 weeks of intervention in 3-month-old (A) and 12-month-old (C) mice, and fat mass (% of body weight) in 3-month-old (B) and 12-month-old (D) mice (n = 6–21/group). (E) Representative H&E image of the epididymal white adipose tissue (eWAT) at collection. (F and G) Serum leptin concentration after 13 weeks of intervention in 12-month-old females (F) and males (G) (n = 12/group). (H) Representative images of F4/80 antibody staining in the eWAT at collection. (I and J) Quantification of adipocytes size (average area in μm^2^) in 3-month-old females and males (I) (n = 4/group) and 12-month-old females and males (J) after 12/13 weeks of intervention (n = 2/group). (K and L) Number of F4/80-stained crown structures in 3-month-old females and males (K) and 12-month-old females and males (L) after 12/13 weeks of intervention (n = 2/group). (M) Representative H&E image of the liver at collection. (N) Liver triglyceride quantification in 12-month-old females and males after 13 weeks of intervention (n = 12/group). (O and P) Serum cholesterol after 12/13 weeks of intervention in 3-month-old females and males (O) and 12-month-old females and males (P) (n = 10–12/group). (Q and R) Serum triglyceride after 12/13 weeks of intervention in 3-month-old females and males (Q) and 12-month-old females and males (R) (n = 10–12/group). Statistics: (B, D, I–L, and N–R) two-way ANOVA (factors: FP and sex; below graph) and Tukey’s multiple comparisons tests (above graph). (F and G) Unpaired t test. Data are presented as mean ± SEM with result of statistical test, with *p < 0.05, **p < 0.01, ***p < 0.001.

To assess the impact of TRF on adiposity, body composition was quantified by small animal MRI at the end of the feeding protocol. Consistent with BW data, there was no difference in body composition between ALF and TRF females at both ages (Figures 2A–2D). Alternatively, in males, TRF was associated with a significant lower fat mass (Figures 2A and 2C), representing 27% versus 17% of the total BW in young males (Figure 2B, adjusted p [adj.p] = 0.0014) and 40% versus 22% in 1-year-old males on ALF and TRF, respectively (Figure 2D, adj.p < 0.0001). Analyzing these results with a two-way ANOVA with FP and sex as biological variables suggests that (i) the effect of both feeding and sex are more pronounced in old mice than in young ones (FP and sex not significant [ns] in young versus FP and sex adj.p < 0.0001 in old) and that (2) the sexual dimorphic effect of TRF increases with age (interaction adj.p = 0.056 in young versus adj.p < 0.001 in old) (Table S1). In 1-year-old males, the reduction in body fat was accompanied by a 44% reduction in serum leptin levels (6.9 ng/mL in M-12mT versus 15.7 ng/mL in M-12mA, p = 0.0162, Figure 2G). Female mice had higher serum leptin level than did the male mice and no differences in leptin levels were observed between ALF or TRF females (Figure 2F).

Histological examination of the epididymal white adipose tissue (eWAT) by H&E staining and quantification of adipocyte size revealed significantly smaller adipocytes (Figures 2E, 2I, and 2J) and fewer inflammatory crown structures in TRF males than in ALF males in both age groups (Figure 2H). Reduced adipose inflammation under TRF was confirmed in males by F4/80 staining and quantification (Figures 2K and 2L). As previously observed (Pettersson et al., 2012), inflammatory crown structures were absent in females irrespective of age or feeding pattern (Figures 2H, 2K, and 2L).

Since the Western diet promotes the development of nonalcoholic fatty liver disease (NAFLD) (Stephenson et al., 2018), we assessed ectopic accumulation of fat in the liver. Histological examination showed fewer lipid droplets in all TRF groups (Figure 2M), and biochemical quantification showed a significant reduction in triglyceride (TG) levels in the liver in both 1-year-old males and females on TRF (Figure 2N). We also assessed serum levels of cholesterol and TG. Although mice fed *ad libitum* a HFD (~60% calories from fat) show higher levels of serum TG (>120 mg/dL) (Chaix et al., 2014, 2019), in this experiment both sexes of mice at both age groups showed <100 mg/dL TG when fed a 45% fat diet ALF. There was no difference in the serum TG levels between any of the ALF versus TRF groups irrespective of sex and age (Figures 2Q and 2R). Serum cholesterol levels were significantly lower in males on TRF irrespective of age (Figures 2O and 2P) but not different in females. Analyzing these results with a two-way ANOVA with FP and sex as biological variables suggests that (1) the effect of the FP is more pronounced in older mice (FP adj.p < 0.01 in young versus adj.p < 0.0001 in old) and (2) that the sex differences increase with age (sex ns in young versus sex adj.p < 0.0001 in old) (Table S1).

In conclusion, ALF feeding of a 45% fat and 17% sucrose diet caused adipose cell expansion and inflammation in males, while both young and older males on TRF were protected from such effects. ALF promoted fatty liver while TRF offered protection to older animals of both sexes. ALF-associated elevated serum cholesterol levels were more pronounced in old males than females, and TRF only imparted a significant reduction in serum cholesterol in males.

### TRF improves glucose regulation irrespective of sex and age

**Figure 3.**
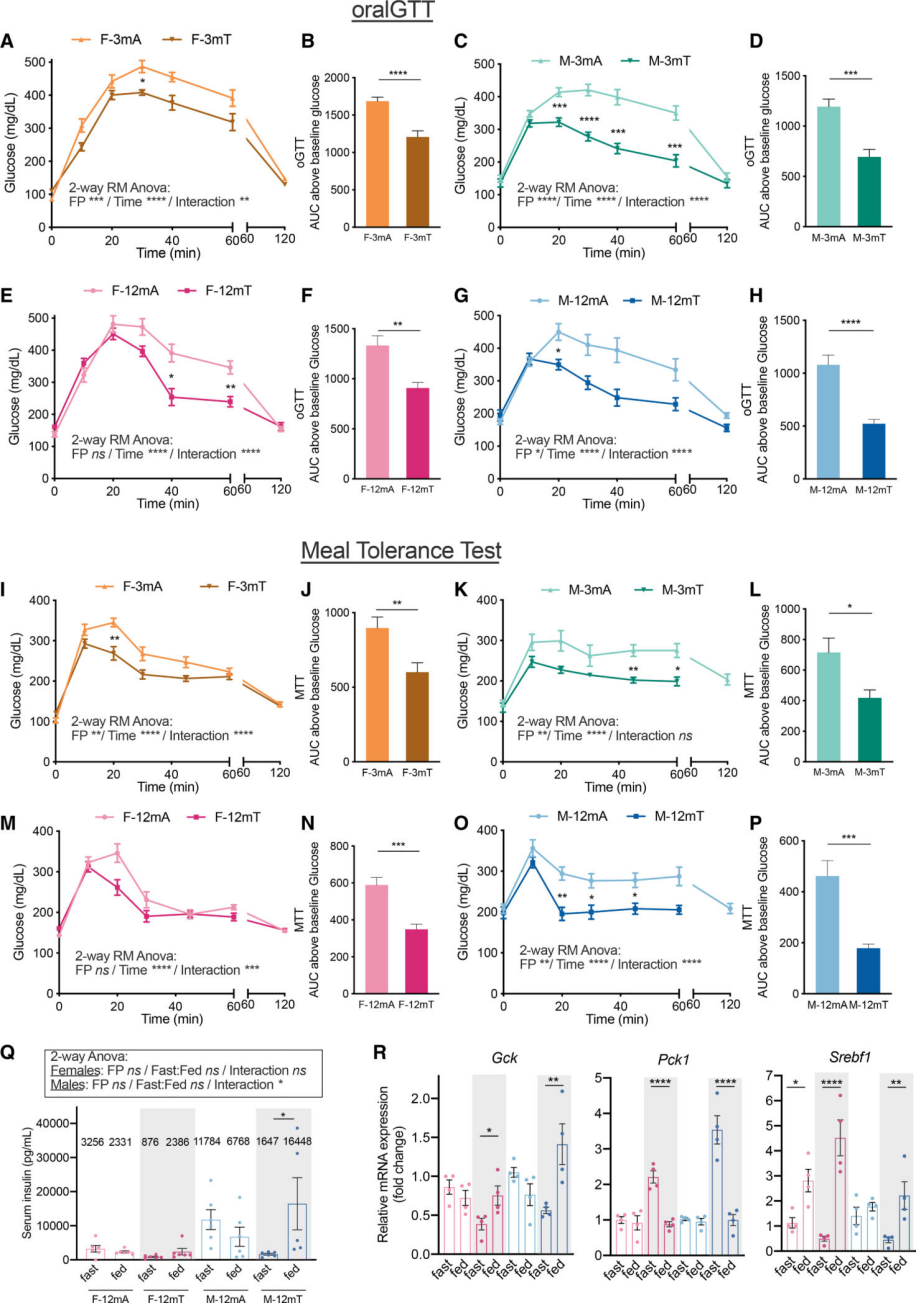
TRF maintains glucose homeostasis in both male and female mice (A–H) Oral GTT. Evolution of blood glucose levels and quantification of the area under the curve (AUC) above baseline glucose after gavage of a constant bolus of 100 mg of glucose after 16 h of fasting (ZT21–ZT13) in (A and B) 3-month-old females (n = 10/group), (C and D) 3-month-old males (n = 10/group), (E and F) 12-month-old females (n = 8–12/group), and (G and H) 12-month-old males (n = 8/group) after 11 weeks of intervention. (I–P) Oral MTT. Evolution of blood glucose levels and quantification of the AUC above baseline glucose after gavage of a constant bolus of 740 calories (approximately 5% of daily intake) of a complete meal (7% protein, 20% carbohydrate, 5% fat) after 16 h of fasting (ZT21–ZT13) in (I and J) 3-month-old females (n = 13–14/group), (K and L) 3-month-old males (n = 8/group), (M and N) 12-month-old females (n = 12/group), and (O and P) 12-month-old males (n = 8/group) after 9 weeks of intervention. (Q) Serum insulin levels in 12-month-old females and males in the subjective fasted state (ZT10) and subjective fed state (ZT18) after 13 weeks of intervention (n = 6/group). (R) Relative mRNA expression (log_2_ fold change) of Gck, PepCK, and Srebf1 in the liver of 12-month-old females and males in the subjective fasted state (ZT12) and subjective fed state (ZT16) after 13 weeks of intervention (n = 4/group). Statistics: (A, C, E, G, I, K, M, and O) Two-way repeated-measures ANOVA (factors: FP and time; inset) and Sidak’s multiple comparisons tests (on graph). (B, D, F, H, J, L, N, and P) Unpaired t test. (Q) Two-way ANOVA (factors: FP and fasted/fed state; inset) and Tukey’s multiple comparisons tests (above graph). (R) Two-way ANOVA for each gene (factors: FP and fasted/fed state) and Sidak’s multiple comparisons tests (above graph). Data are presented as mean ± SEM with result of statistical test, with *p < 0.05, **p < 0.01, ***p < 0.001.

Obesity resulting from the consumption of a high-fat high-sucrose diet is a major risk factor for the development of glucose intolerance and insulin resistance (Kahn and Flier, 2000; Kahn et al., 2006). At the end of the feeding protocol, oral glucose tolerance tests (oGTTs) (Figures 3A–3H) were sperformed with a bolus of 100 mg of glucose after 16 h of fasting (ZT21–ZT13). TRF was associated with a lower increase in blood glucose and a faster return to normoglycemia in both 3-month-old (Figures 3C and 3D) and 12-month-old (Figures 3G and 3H) males. Interestingly, the oGTT response curve was identical between young and old males, suggesting that the effect of the diet was outweighing the effect of age on glucose intolerance. There was also a significant improvement in glucose tolerance in 3-month-old (Figures 3A and 3B) and 12-month-old (Figures 3E and 3F) female mice on TRF. Similarly, middle-aged females and males on TRF were able to restore normoglycemia more efficiently than ALF mice upon an intraperitoneal (i.p.) GTT (ipGTT) (Figures S2A–S2C).

In order to assess whether TRF impacted whole-body glucose homeostasis upon feeding, we performed a meal tolerance test (MTT) in which mice were gavaged with a complete liquid meal (Figures 3I–3P). All mice received a constant bolus of 740 cal (approximately 5% of daily intake) of a meal comprised of 29% calories from fat, 58% from carbohydrate, and 15% from protein (Ensure Plus), after 16 h of fasting (ZT21–ZT13). In general, normoglycemia after MTT was restored faster than after oGTT for the same amount of sugar ingested. This is likely due to increased engagement of the incretin response by other macronutrients such as lipids and amino acids (Ezcurra et al., 2013). In all groups of 3- and 12-month-old females (Figures 3I, 3J, 3M, and 3N) and males (Figures 3K, 3L, 3O, and 3P), TRF was associated with a significant improvement in blood glucose regulation after a meal challenge. Remarkably, normoglycemia was reached faster in males than in females.

Finally, in order to assess insulin sensitivity, we measured insulin levels in 1-year-old females and males (Figure 3Q) in their natural feeding state at two different times of day: ZT10, during the light phase, representing the “subjective fast”; and ZT18, in the middle of the dark phase, representing the “subjective fed” state. Compared to males, the older female mice had lower insulin levels in all conditions. TRF had a modest effect on reducing the fasted insulin in older females. Insulin levels were constantly elevated in M-12mA, a marker of insulin resistance, whereas they were significantly reduced during the “fasted” state in M-12mT, suggesting improved insulin sensitivity (Figure 3Q). These results were confirmed by a significant reduction in the homeostasis model assessment of insulin resistance (HOMA-IR) (Figure S2D) in old males on TRF.

Since both sexes were protected from fatty liver, we hypothesized that hepatic suppression of gluconeogenesis could be responsible for faster glucose regulation. We thus assessed mRNA expression of key enzymes and regulators Gck, Pepck, and Srebf1. In 1-year-old females and males on TRF, feeding was associated with a significant increase in mRNA expression of Gck and Srebf1, and a significant decrease in mRNA expression of Pepck (Figure 3R). These changes in GCK expression were confirmed at the protein level in males (Figure S2F). These results suggest that TRF is associated with increased hepatic glucose utilization and suppression of gluconeogenesis upon feeding, indicating improved metabolic flexibility. In conclusion, TRF preserves whole-body glucose regulation and protects from markers of insulin resistance in both males and females irrespective of age.

### TRF protects muscle mass and improves muscle performance in a sex-dependent manner

**Figure 4.**
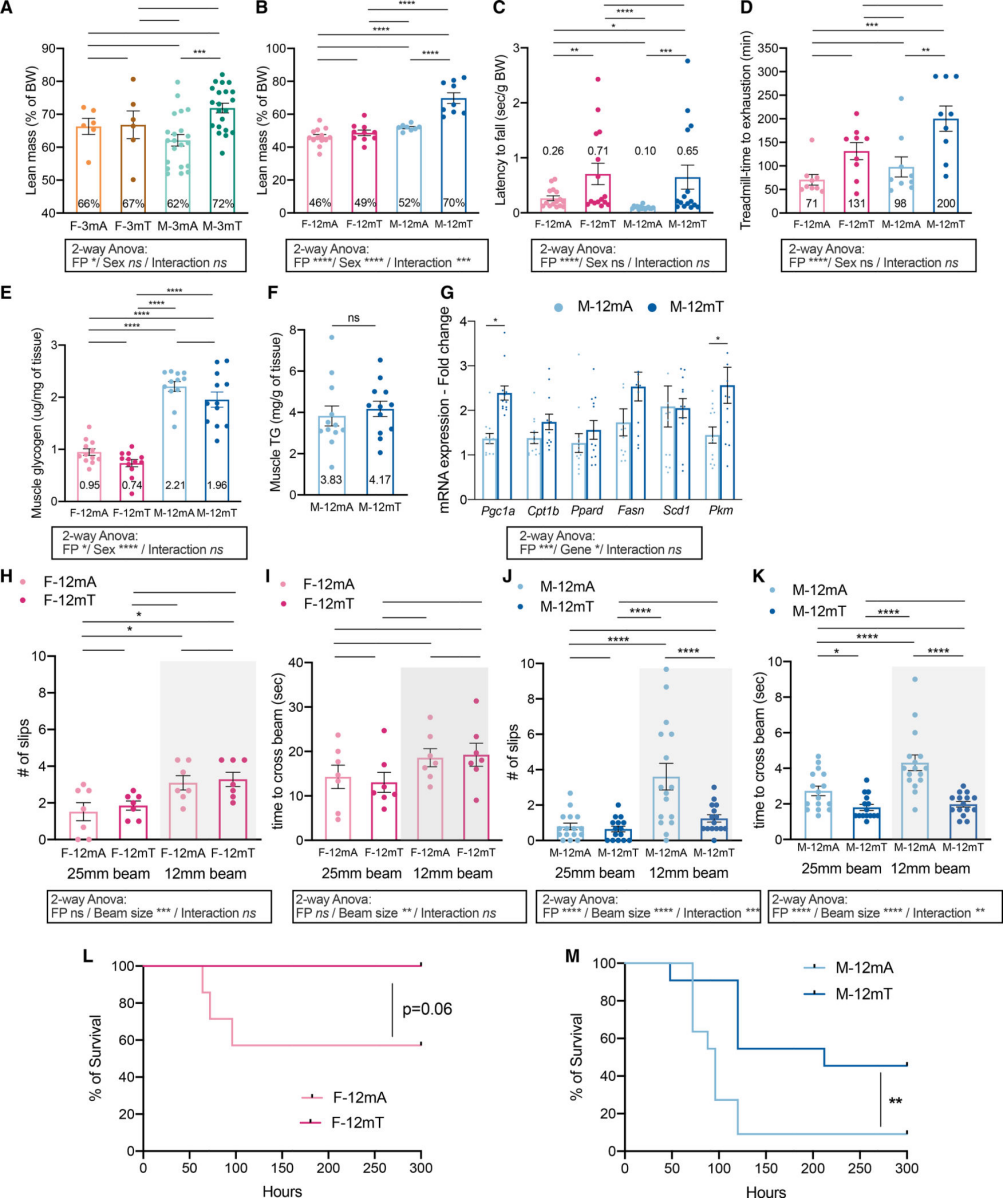
Sex-specific effects of TRF on physical performance and response to LPS challenge (A and B) Lean mass (% of body weight) after 10 weeks of intervention in 3-month-old (A) and 12-month-old (B) females and males (n = 6–21/group). (C) Wire hang test performance in 12-month-old female and male mice after 11 weeks of intervention represented as the time to fall normalized to body weight (n = 15/group). (D) Performance in a treadmill run-to-exhaustion assay in 12-month-old female and male mice after 11 weeks of intervention (n = 9/group). (E) Muscle glycogen content in 12-month-old females and males after 13 weeks of intervention (n = 12/group). (F) Muscle triglyceride content in 12-month-old males after 13 weeks of intervention (n = 12/group). (G) mRNA expression of fat and glucose metabolism genes Pgc1a, Cpt1b, Ppard, Fasn, Scd1, and Pkm in the muscle of 12-month-old males after 13 weeks of intervention (n = 12/group). (H–K) Performance (number of slips and time to cross) in a beam crossing assay in (H and I) 12-month-old females (n = 7/group) and (J and K) males after 12 weeks of intervention (n = 15–16/group). (L and M) Survival curve of (L) 12-month-old females subjected to a LPS challenge of 5 μg (n = 7/group) and (M) 12-month-old males subjected to a LPS challenge of 10 μg (n = 11/group) after 13 weeks of intervention. Statistics: (A–E) Two-way ANOVA (factors: FP and Sex; below graph) and Tukey’s multiple comparisons tests (above graph). (F) Unpaired t test. (G) Two-way ANOVA (factors: FP and gene; below graph) and Sidak’s multiple comparisons tests (above graph). (H–K) Two-way ANOVA (factors: FP and beam size; below graph) and Tukey’s multiple comparisons tests (above graph). (L and M) Log-rank (Mantel-Cox) test. Data are presented as mean ± SEM with result of statistical test, with *p < 0.05, **p < 0.01, ***p < 0.001.

Body composition analysis revealed that the lean mass as a percentage of BW was similar in male and female mice fed ALF within the same age group. However, under TRF, a significantly higher lean mass relative to ALF was observed only among males both at young (72% in M-3mT versus 62% in M-3mA, adj.p = 0.0007, Figure 4A) and older age (70% in M-12mT versus 52% in M-12mA, adj.p < 0.0001, Figure 4B) (Table S1). Interestingly, the older mice of both sexes under ALF had reduced lean mass compared to younger mice. Yet, the similar percentage of lean mass in both 3- and 12-month-old male mice on TRF suggests that TRF might be an efficient strategy against aging-associated muscle loss. The observed differences in lean mass prompted us to test for differences in muscle strength and endurance in 1-year-old mice. In a wire hang test, both M-12mT and F-12mT were able to hang by their four limbs for significantly longer than M-12mA and F-12mA (28 and 24 s on average for old females and males on TRF, respectively, versus 13 and 5 s for females and males, respectively) (Figure S4A), suggesting better muscle function. Importantly, these conclusions are maintained when normalized to BW (Figure 4C; Table S1). There was no difference in forelimb grip strength in middle-aged males and females on ALF or TRF (Figure S3B). M-12mT also ran for significantly longer in a treadmill run-to-exhaustion assay (200 min on average for M-12mT versus 98 min for M-12mA, adj.p = 0.0056; Figure 4D). Although the female mice under TRF showed some improvement relative to their ALF counterparts, the increase did not reach significance.

To explore the biochemical and molecular determinants of these differences in muscle function, we measured muscle fuel sources and expression of mRNAs encoding key muscle metabolic regulators. We did not detect differences in muscle glycogen or TG content (Figures 4E and 4F; Table S1) between ALF and TRF groups, suggesting that differences in total fuel availability may not be responsible for the observed differences in performance in male mice. There was no difference in mRNA expression of fatty acid oxidation rate-limiting enzyme Cpt1b or Ppard or in lipogenic enzyme Fasn and Scd1. There was a significant increase in mRNA expression of the glycolytic enzyme Pkm that could suggest higher glucose utilization in TRF muscle as well as a significant increase in Pgc1α (Figure 4G). PGC-1α is the master transcription regulator that stimulates mitochondrial biogenesis, which could underlie the observed differences in muscle mass and function upon TRF.

We also assessed motor coordination in 1-year-old mice using a raised beam crossing challenge. In this assay the mice have to coordinate their limbs to walk on a 25- or 12-mm-wide beam (Bourane et al., 2015). There was no difference in the number of paw slips or time to cross on the control 25-mm-wide beam in 1-year-old females (Figures 4H and 4I; Figures S3D and S3E; Table S1) or males (Figures 4J and 4K; Figures S3D and S3E; Table S1). When challenged with a narrower 12-mm-wide beam that requires more sensory motor coordination, we observed no differences in females (Figures 4H and 4I), but M-12mT performed significantly better than M-12mA with fewer paw slips (Figure 4J) and a shorter duration to cross (Figure 4K). There was no difference in the latency to fall from the rod in middle-aged males and females on ALF or TRF when challenged on an accelerating rotating rod (Figure S3C). These results suggest that 1-year-old males on TRF performed better in a challenging motor task than did those on ALF. In conclusion, in comparison to ALF, TRF preserves muscle mass, function, and performance as well as motor coordination in 1-year-old males but not in females.

### TRF increases survival to LPS septic challenge

Sepsis leading to multi-organ failure is a highly lethal condition that remains a significant clinical challenge in intensive care units as exemplified recently during the SARS-CoV-2 pandemic (Cohen et al., 2015). Pre-existing metabolic disease and overall health of organs in the body can dramatically influence resilience to infectious diseases in general and to sepsis (Ayres, 2020; Bornstein et al., 2020). Since TRF appears as a multi-solving intervention that imparts pleiotropic benefits to multiple organs, we set out to determine whether middle-aged mice on TRF would have better survival outcome upon a septic challenge. We used exogenous administration of endotoxin (LPS treatment) to induce a mid-grade systemic inflammatory condition to mimic a pre-sepsis condition and followed survival.

A preliminary experiment suggested a sex difference in septic shock response, with the lethal dose 50 (LD_50_) in females being lower than in males (see STAR Methods). Accordingly, we used a constant i.p. dose of 5 μg of LPS in female mice and a constant i.p. dose of 10 μg of LPS in males. Survival was monitored for up to 13 days following LPS challenge. In 1-year-old males, TRF was associated with a significant increase in the percentage of survival to LPS (n = 11/group, p = 0.0082, Figure 4M). For 1-year-old females, none of the female TRF mice succumbed to LPS challenge and there was a non-significant trend to increased survival (n = 7/group, p = 0.0597, Figure 4L). In conclusion, TRF can increase survival to sepsis in middle-aged mice.

### Limitations of the study

Therefore, the findings in this study from the C57BL6/J strain of mice may not be representative of other rodent strains, yet these results offer an experimental framework for mechanistic studies. Our use of 3 months of TRF is in line with several rodent and human studies using such an intervention. It is likely that a longer duration of TRF intervention might increase the magnitude of differences between ALF and TRF groups. This may be specifically important for several assays for female mice where we did not find a statistically significant difference, but there was a trend similar to that found among males. Nevertheless, the results imply that the rate of metabolic changes under TRF is modulated by sex.

There are also limitations of this preclinical animal model for human TRE studies. The response to TRE in the heterogeneous human population may differ from that in inbred mouse species. Furthermore, in many TRE studies in humans, there is an inadvertent decrease in energy intake, which may contribute to reported weight loss in both sexes in many TRE pilot studies. Despite these limitations, the study offers a powerful framework to assess the impact of an obesogenic diet and of TRF intervention in both sexes and at two different ages.

## Conclusions

Our study provides a parallel systematic evaluation of the metabolic and performance effect of 3 months of Western diet feeding in young and middle-aged male and female C57BL/6J mice in standard *ad libitum* feeding conditions and under TRF dietary intervention. The age of the mice, precise composition of the diet, and location of the animal study critically contribute to metabolic parameters (Corrigan et al., 2020). In this study, all groups were analyzed in the same facility with the same research staff, with a sole source for the diet (Research Diet D12451) and the mice (The Jackson Laboratory), and will thus serve as a fundamental resource for researchers across the nutrition, exercise, metabolic, and circadian research community.

There are several key conclusions from this study. In agreement with previous studies, we show a sexually dimorphic response to Western diet feeding between males and females, with reduced insulin levels and adipose tissue inflammation in females. Nevertheless, females, especially as they aged, were still glucose intolerant and highly sensitive to fatty liver. Dietary intervention such as TRF was able to protect the female mice from these metabolic impairments. Alternatively, no benefits of TRF were seen in performance and behavior assays in females, suggesting that additional interventions might be necessary to improve these health outcomes in females. In males, TRF improved all metabolic and performance parameters tested, regardless of age, suggesting that TRF could improve health-span and lifespan even with Western diet feeding. Future studies of lifelong TRF will test this hypothesis. Finally, we show that being on TRF can increase survival to a septic challenge in middle-aged male mice, with a trend in females. This is especially relevant in the context of the COVID-19 pandemic during which this paper was written since poor metabolic health is the major risk factor for severe COVID-19 (Ayres, 2020; Bornstein et al., 2020). Future studies will determine whether TRF could also improve metabolic health complications that have been observed in survivors of previous SARS-CoV-2 infections.

## STAR★METHODS

### RESOURCE AVAILABILITY

#### Lead contact

Further information and requests for resources and reagents should be directed to and will be fulfilled by the lead contact, Satchidananda Panda (satchin@salk.edu).

#### Materials availability

This study did not generate new unique reagents.

#### Data and code availability

The summary statistics for all data presented in the manuscript is available in the supplemental table. Data presented in this manuscript will be shared by the lead contact upon request.

The manuscript does not report any new code.

Any additional information required to reanalyze the data reported in this paper is available from the lead contact upon request.

### EXPERIMENTAL MODEL AND SUBJECT DETAILS

#### Animals, diets, and experimental cohorts

All animal experiments were carried out in accordance with the guidelines and approved by the IACUC of the Salk Institute. All C57BL/6J mice were purchased from The Jackson Laboratory (stock 000664) and the diet from Research Diet (D12451). All mice were entrained to a 12h light:12h dark cycle with normal chow food available *ad libitum* for 2 weeks before being randomly assigned (with equal body weight at start in each group) to the *ad libitum* group (ALF or A) or the time restricted feeding group (TRF or T). The TRF group had access to food for 9 hours during the dark active phase, from ZT13 to ZT22 where ZT0 denotes light on. Food intake and body weight were monitored weekly throughout the 12 weeks experiments. The feeding protocol was repeated in two independent animal cohorts except in 3 months old females.

### METHOD DETAILS

#### Body composition

Body composition was analyzed in live mice using a body composition analyzer (EchoMRI™−100H).

#### Indirect calorimetry, food intake, and activity

Food intake, locomotor activity, oxygen consumption, and carbon dioxide production were simultaneously measured for individually housed mice with a LabMaster system (TSE Systems). Mice were acclimatized for 2–3 days, data were collected for 4–5 days and analyzed. Light and feeding conditions were kept the same as in the home cages.

#### Sample collection

After 12 weeks on the feeding paradigm, animals were sacrificed and blood, liver, epidydimal white adipose tissue (eWAT) and muscle samples collected. One piece of organ was flash frozen, ground to fine powder in liquid nitrogen and stored at −80C for further analysis. Another piece was fixed in formalin for histology.

#### Hepatic and muscle triglycerides and glycogen quantification

Accurate weights of frozen liver and muscle powder were homogenized in 5% NP40 and water for TG and glycogen measurements respectively. Triglyceride and glycogen concentration were measured using enzymatic assays (Triglycerides LiquiColor, Stanbio & Glycogen Assay Kit, Sigma) according to the manufacturer’s instruction. Data were normalized to weight.

#### Histology

6 μm sections of formalin-fixed liver and epididimal WAT (eWAT) were stained with H&E; 10 μm sections of eWAT were stained with F4/80. Slides were imaged under a light microscope. Adipocyte cell size (area in μm^2^) was quantified (Adiposoft ImageJ plug-in) (Galarraga et al., 2012) in 4 sections per mouse (approx. area per section is 2 mm^2^ and approx. number of adipocytes per section is 300) using 4 mice per group for 3 months old mice and 2 mice per group for 12 months old mice. The number of F4/80 stained Crown structures were manually counted in 6 sections per mouse (approx. area per section is 2 mm^2^ and approx. number of adipocytes per section is 300) in 2 mice per group. F4/80 staining intensity was also measured using ImageJ color devolution for DAB and measuring mean gray area. The 2 methods produced identical statistical differences results.

#### Serum biochemistry

Glucose, triglycerides and total cholesterol were measured using Thermo Scientific Infinity Reagents. Insulin and leptin were quantified by ELISA (Crystal Chem). HOMA-IR was calculated as follows: (fasting serum insulin concentration (mU/ml)) x (fasting blood glucose levels (mg/dl))/(405) (Matthews et al., 1985).

#### Glucose and meal tolerance tests (ipGTT, oGTT, and MTT)

Mice were fasted for 16 hours from ZT21-ZT13. For ipGTT, mice were ip-injected with 1.5g/kg BW. For oGTT, mice were gavaged with a constant bolus of 100 mg of glucose (independently of their body weight). For MTT, mice were gavaged with a constant meal bolus of 740 cal (approximately 5% of daily intake; Ensure Plus: 29% calories from fat - 58% from carbohydrate - 15% from protein). Blood glucose level was measured using novaMax Plus glucose meter prior to injection and several times after injection as indicated.

#### Performance assays

Rotarod performance test was performed using the Rotamex rotarod (Columbus Instruments) starting at ZT16. On day 1 (training), first, mice were given 3 attempts to maintain position on non-rotating rod up to 60 s. Second, mice were given 2 attempts to balance on rotating (3rpm) but nonaccelerating rod up to 60 s. Third, mice were given one attempt to balance on rotating (3rpm) but nonaccelerating rod up to 60 s and the time to fall during this attempt was recorded as training values. On day 2 (testing), mice were placed on an accelerating rotating rod (0–300rpm) and the time to fall was recorded. Mice were trained for 1 day and performance was recorded on day 2. Data represent the average time spent on the rod for 5 trials on day 2.

The treadmill exhaustion test was performed using the Exer 6M first generation treadmill (Columbus Instruments) starting at ZT16. On day 1, mice were trained at low speed (6 m/min – 8 m/min) for 15 minutes with speed ramping up every 5 minutes. On day 2, mice were trained for 15 minutes at intermediate speed (8 m/min – 10 m/min) with 5 minutes of ramping. On day 3, maximal speed (12 m/min) was reached by ramping up every 5 minutes. 5 degree angle of incline was applied to the treadmill when running time exceeded 1 hour and 10 degree angle of incline was applied when running time exceeded 1 hour 30 minutes. Mice were run until exhaustion, defined as the inability to continue running despite repeated stimulation by brushes and compressed air. Runs were stopped after 290 minutes.

Grip strength was determined using a digital grip strength meter (Columbus Instruments) at ZT16. Each animal was tested three times. Experimenter was blind to the feeding group.

Kondziela’s inverted screen test was performed as described (Deacon, 2013) starting at ZT16. Mice were placed on a flat wire-mesh screen, screen was then inverted such that mice have to use all 4 limbs to hang on to the screen and time to fall off was recorded. Each mouse had 3 trials. Results are represented as the latency to fall normalized to body weight (sec/g BW).

Raised beam crossing test was performed on a custom-made experimental setup (Salk Behavioral Testing Core) starting at ZT15. Mice were recorded using cameras positioned at 2 different angles while crossing raised (50 cm above tabletop) beams (25 mm or 12 mm wide square beam, 1 m length). Mice were recorded for 3 consecutive trials on the 25mm and the 12mm beam separated by a 30 min break. The numbers of foot slips and the time to cross the beams were scored for each animal.

#### qRT-PCR

RNA was prepared using Trizol extraction. cDNA was obtained using qScript cDNA synthesis Kit (Quanta bio). qRT-PCR was performed using 40ng of liver cDNA and 500nM primer mix per reaction. Results were normalized to Hprt1 and analyzed using the DDCt methods. Primer sequences can be found in the key resource table.

#### Western blotting

Total liver and muscle lysates were prepared in RIPA buffer (20 mM Tris-HCl (pH 7.5), 150 mM NaCl, 1 mM EGTA, 1% NP-40, 1% sodium deoxycholate, 1 mM Na3VO4) supplemented with protease and phosphatase inhibitor cocktails (Complete and PhosSTOP tablets, Roche). Membranes were probed with indicated antibodies (see resource table for ref.).

#### LPS challenge

In order to determine the dose of LPS to inject (LD50), a pilot experiment was conducted in 1-year-old obese male and female mice. The LPS dose was chosen based on (1) the induction of severe symptoms in 100% of the mice (labored breathing, little response to stimulus, and complete inactivity after 36h) and (2) the survival of 50% of the mice 4 days post injection. Female mice received a constant IP dose of 5 μg, whereas males received a constant IP dose of 10 μg. During the challenge, all mice were single-housed and had *ad libitum* access to food. Health status was monitored twice daily and animals’ life terminated and recorded as time of death according to predefined humane endpoints.

### QUANTIFICATION AND STATISTICAL ANALYSIS

A description of the statistical tests used for each figure panel and their results is provided in Table S1.

Two-way Repeated-measures (RM) ANOVA (feeding paradigm (FP) x Time) followed by Sidak’s multiple comparisons test was used for time-series experiments.

Two-way ANOVA with interaction followed by Tukey’s multiple comparisons test was used when analyzing the effect of (feeding paradigm (FP) x sex) or (feeding paradigm (FP) x fed:fasting state) or (feeding paradigm (FP) x gene) or (feeding paradigm (FP) x beam size) or (feeding paradigm (FP) x age).

One-way ANOVA was used when analyzing the effect of the feeding paradigm (FP) in more than 2 groups.

Unpaired two-tailed t test was used when analyzing the effect of the feeding paradigm (FP) in 2 groups.

Statistics were calculated using GraphPad Prism 6.0. Unless otherwise noted, throughout all figures, data are presented as mean ± SEM with statistical result of the statistical test, with *p < 0.05, **p < 0.01, ***p < 0.001. Statistical parameters, including the value of n, are noted in figure legends. Statistical significance was concluded at p < 0.05. Indirect calorimetry data were also analyzed using the CalrApp (Mina et al., 2018).

## Supplementary Material

1

2

## Figures and Tables

**KEY RESOURCES TABLE:** 

REAGENT or RESOURCE	SOURCE	IDENTIFIER
Antibodies		
F4/80	Cell Signaling Technology	RRID:AB_2799771
GCK	Santa Cruz Biotechnology	RRID:AB_2107620
Chemicals, peptides, and recombinant proteins		
D-Glucose	Sigma-Aldrich	G8270
Lipopolysaccharides from *Escherichia coli* O111:B4	Sigma-Aldrich	L3024
Critical commercial assays		
Infinity Glucose Hexokinase	Thermo Scientific	TR15421
Infinity Cholesterol	Thermo Scientific	TR13421
Infinity Triglycerides	Thermo Scientific	TR22421
Stanbio Triglycerides LiquiColor Test (Mono)	Fisher Scientific	SB2200225
Mouse Metabolic Kit	Meso Scale Diagnostics	K15124C
Experimental models: Organisms/strains		
C57BL/6J	The Jackson Laboratory	000664
Oligonucleotides		
Mm_Gck_Fwd	This study	AACGACCCCTGCTTATCCTC
Mm_Gck_Rev	This study	CTGCCAGGATCTGCTCTACC
Mm_Pck1_Fwd	This study	CTGAAGGTGTCCCCCTTGTC
Mm_Pck1_Rev	This study	GATCTTGCCCTTGTGTTCTGC
Mm_Srebf1_Fwd	This study	CTTTTCCTTAACGTGGGCCT
Mm_Srebf1_Rev	This study	GAGCTGGAGCATGTCTTCGAT
Mm_Pgc1a_Fwd	This study	GAAAGGGCCAAACAGAGAGA
Mm_Pgc1a_Rev	This study	GTAAATCACACGGCGCTCTT
Mm_Cpt1b_Fwd	This study	CCTGGTGCTCAAGTCATGGT
Mm_Cpt1b_Rev	This study	CCTGGTGCTCAAGTCATGGT
Mm_Ppard_Fwd	This study	CAAACCCACGGTAAAGGCAG
Mm_Ppard_Rev	This study	TGGCTGTTCCATGACTGACC
Mm_Fasn_Fwd	This study	CGGATTCGGTGTATCCTGCT
Mm_Fasn_Rev	This study	CCTCGGGTGAGGACGTTTAC
Mm_Scd1_Fwd	This study	GTGCCGTGGGCGAGG
Mm_Scd1_Rev	This study	AGCCCAAAGCTCAGCTACTC
Mm_Pkm_Fwd	This study	CCACACAGATGCTGGAGAGC
Mm_Pkm_Rev	This study	TTCAAACAGCAGACGGTGGA
Software and algorithms		
ImageJ	NIH	https://imagej.nih.gov/ij/
Prism 6.0	GraphPad Software	https://www.graphpad.com
Other		
Rodent Diet With 45 kcal% Fat	Research Diets, Inc	D12451
Criterion TGX Stain-Free Protein Gel	Bio-Rad	5678035

## Highlights

- TRF protects against body weight gain in young and old male mice but not in female mice
- TRF protects from fatty liver regardless of sex or age
- TRF preserves whole-body glucose regulation in males and females irrespective of age
- TRF extends muscle performance, motor coordination, and survival to sepsis in old mice
