# Supplementary material for: Sex- and age-dependent outcomes of 9-hour time-restricted feeding of a Western high-fat high-sucrose diet in C57BL/6J mice
Source: Cell Rep. Author manuscript; Available in PMC 2021 Oct 8. (PMC8500107; doi:10.1016/j.celrep.2021.109543)
Supplement: 1 [file NIHMS1734073-supplement-1.pdf]

Cell Reports, Volume *36*

Supplemental information

Sex- and age-dependent outcomes of 9-hour time-restricted feeding of a Western high-fat high-sucrose diet in C57BL/6J mice

Amandine Chaix, Shaunak Deota, Raghav Bhardwaj, Terry Lin, and Satchidananda Panda

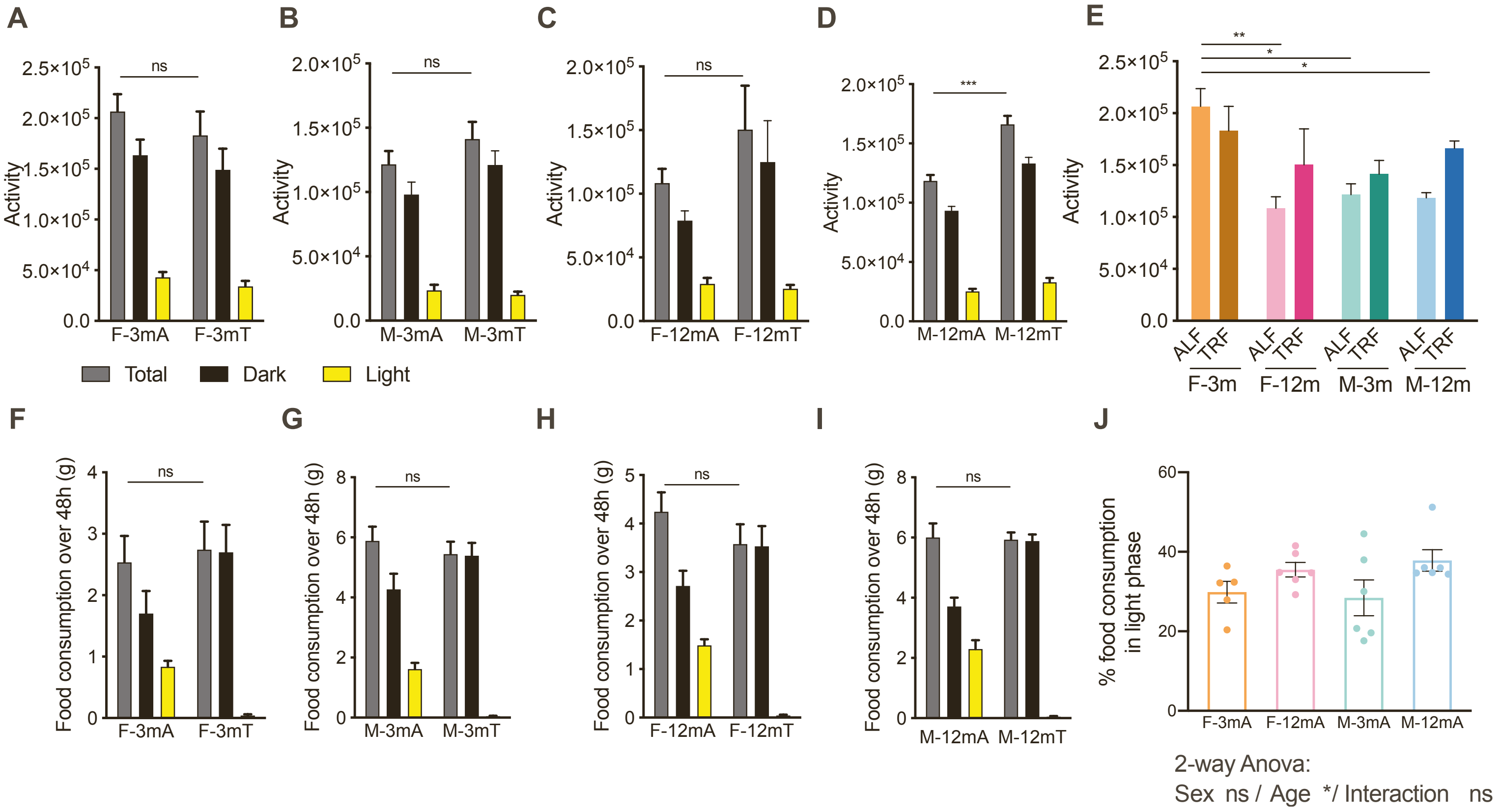

Figure S1, related to Figure 1: Activity levels and food consumption for all mice groups under study.
(A-D) Total activity (grey), as well as dark:light repartition over 48h of recording in metabolic chambers in 3 months old females (A) and males (B), and 12 months old females (C) and males (D) after 10 weeks of intervention (n=5-6/group). (E) Total activity in all groups of mice (n=5-6/group). (F-I) Total food consumed (in g) (grey), as well as dark:light repartition over 48h of recording in metabolic chambers in 3 months old females (F) and males (G), and 12 months old females (H) and males (I) after 10 weeks of intervention (n=5-6/group). (J) Percentage of food consumption during the light phase in ad lib fed mice (from Fig.1C-F).
Statistics: (A-D), (F-I): Unpaired t-test. (E) Two-way ANOVA (Factors: Group & Feeding Paradigm (FP) and Tukey's multiple comparisons tests (above graph). (J) One-way ANOVA and Sidak's multiple comparisons tests (above graph).

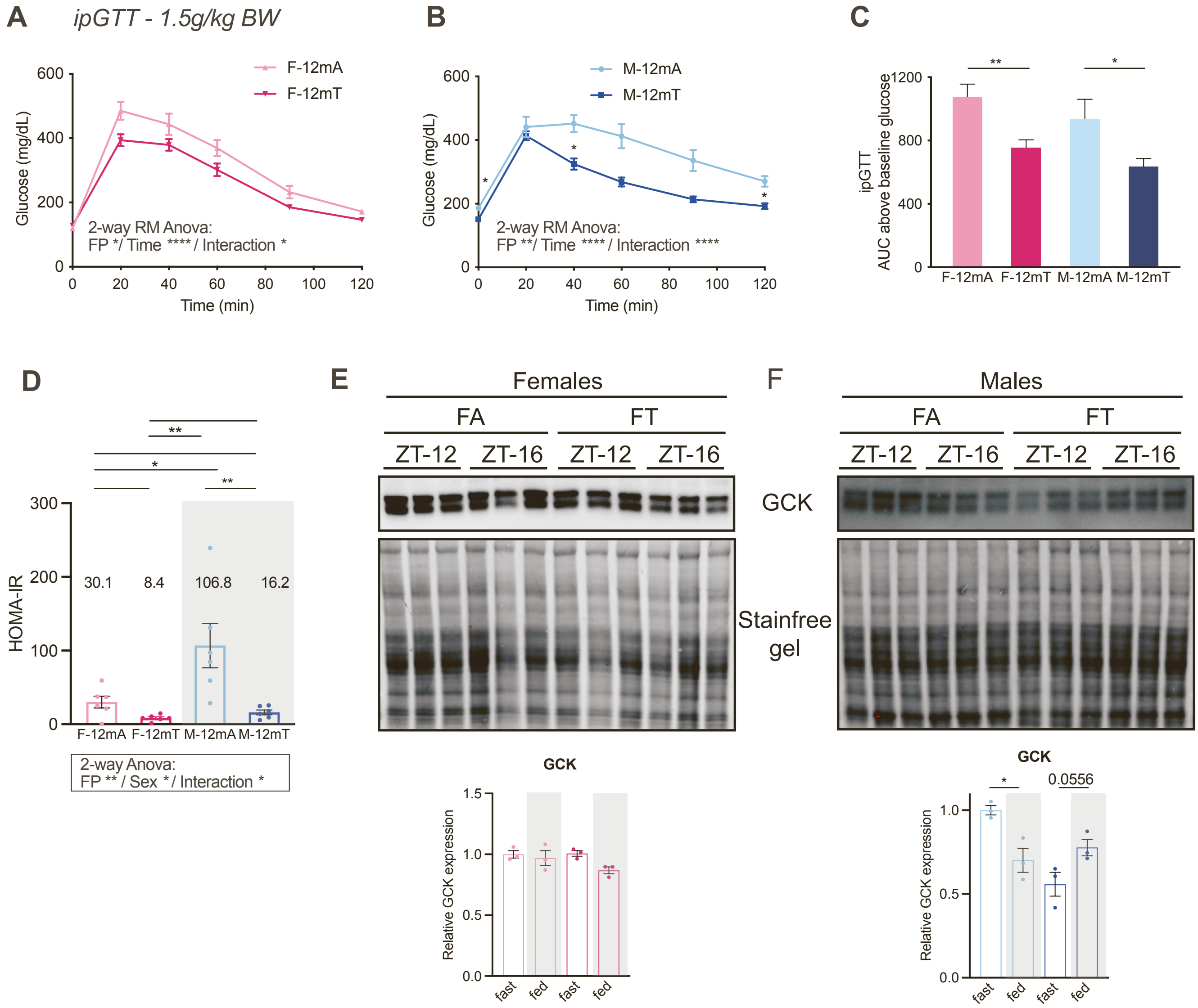

Figure S2, related to Figure 3: TRF improves glucose regulation irrespective of sex and age.
(A-C) ipGTT. Evolution of blood glucose levels (A-B) and quantification of the AUC above baseline glucose (C) after ip injection of 1.5g/kg of BW of glucose after 16h of fasting (ZT21-ZT13) in (A) 12 months old females (n=6-8/group), and (B) 12 months old males after 11 weeks of intervention (n=17-9/group). (D) HOMA-IR in 12 months old females and males after 11 weeks of intervention (n=6/group). (E-F) GCK protein expression and relative quantification (below) in (E) 12 months old females, and (F) 12 months old males in the subjective fasted state (ZT12) and subjective fed state (ZT16) after 13 weeks of intervention (n=3/group).
Statistics: (A-B) Two-way Repeated Measures ANOVA (Factors: Feeding Paradigm (FP) & Time; inset) and Sidak's multiple comparisons tests (above graph). (C) Unpaired t-test. (D) Two-way ANOVA (Factors: Feeding Paradigm (FP) & Sex, below graph) and Tukey's multiple comparisons tests. (E-F) Two-way ANOVA (Factors: Feeding Paradigm (FP) & Fasted:Fed state) and Sidak's multiple comparisons tests (above graph).

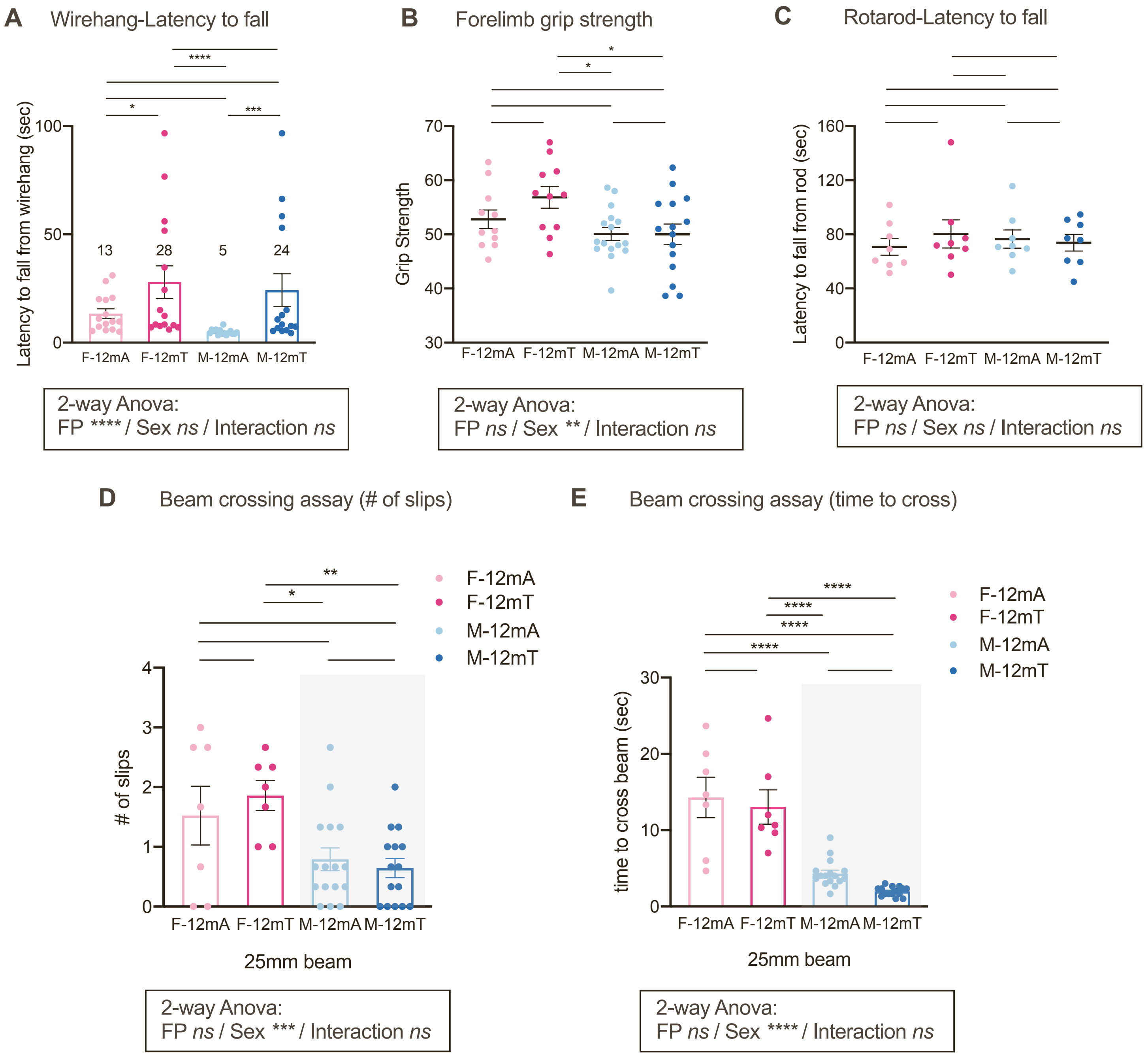

Figure S3, related to Figure 4: TRF regulates muscle performance in a sex-dependent manner.
(A) Wire hang test performance in 12 months old female and male mice after 11 weeks of intervention represented as the time to fall (n=15/group). (B) Forelimb grip strength in 12 months old female and male mice after 11 weeks of intervention (n=11-16/group). (C) Latency to fall (sec) from a rotating rod in 12 months old female and male mice after 10 weeks of intervention (n=8/group). (C-E) Performance on the 25mm beam (number of slips (C) and time to cross (D)) in a beam-crossing assay in 1
2 months old females (n=7/group) and males after 12 weeks of intervention (n=15-16/group). Same data as in Fig. 4H,K but grouped differently to test for the effect of sex.
Statistics: (A-E) Two-way ANOVA (Factors: Feeding Paradigm (FP) & Sex, below graph) and Tukey's multiple comparisons tests (above graph).
